# Molecular Therapies for Inherited Retinal Diseases—Current Standing, Opportunities and Challenges

**DOI:** 10.3390/genes10090654

**Published:** 2019-08-28

**Authors:** Irene Vázquez-Domínguez, Alejandro Garanto, Rob W. J. Collin

**Affiliations:** Department of Human Genetics and Donders Institute for Brain, Cognition and Behaviour, Radboud University Medical Center, 6525GA Nijmegen, The Netherlands

**Keywords:** inherited retinal diseases, IRD, DNA therapies, RNA therapies, compound therapies, clinical trials

## Abstract

Inherited retinal diseases (IRDs) are both genetically and clinically highly heterogeneous and have long been considered incurable. Following the successful development of a gene augmentation therapy for biallelic *RPE65*-associated IRD, this view has changed. As a result, many different therapeutic approaches are currently being developed, in particular a large variety of molecular therapies. These are depending on the severity of the retinal degeneration, knowledge of the pathophysiological mechanism underlying each subtype of IRD, and the therapeutic target molecule. DNA therapies include approaches such as gene augmentation therapy, genome editing and optogenetics. For some genetic subtypes of IRD, RNA therapies and compound therapies have also shown considerable therapeutic potential. In this review, we summarize the current state-of-the-art of various therapeutic approaches, including the pros and cons of each strategy, and outline the future challenges that lie ahead in the combat against IRDs.

## 1. Introduction

### 1.1. Inherited Retinal Diseases (IRDs)

Inherited retinal diseases (IRDs) are a rare and heterogeneous group of neurodegenerative disorders that collectively result in progressive visual impairment. IRDs are estimated to affect around 1 in 2000 people worldwide [1]. Over 250 causative genes have been identified in which mutations can cause one or more of the clinical subtypes of IRD (https://sph.uth.edu/retnet/). IRDs can be familial or sporadic, isolated (non-syndromic) or syndromic and stationary or progressive. In terms of geographic distribution, they could be diffused or localized. Most forms of IRD mainly affect photoreceptors but other forms can also primarily affect the retinal pigment epithelium (RPE) or the inner retina. IRDs can propagate through all modes of inheritance—autosomal dominant (AD), autosomal recessive (AR), X-linked (XL) or mitochondrial, whilst digenic cases or uniparental disomy have also been described occasionally [2].

### 1.2. Present Treatment of IRD

The eye is an ideal target for molecular therapies, for various reasons. First, the tight junctions of the blood-retina barrier (BRB) define the retina to be a relatively immune-privileged tissue [3]. In other words, the introduction of a foreign antigen (like a viral vector) is generally well tolerated without evoking severe inflammatory responses [4,5]. The risk of widespread dissemination of the locally administered vector is low, preventing unwanted systemic effects. Furthermore, relatively small amounts of the vector are needed to achieve a therapeutic response [6,7]. The eye is easily accessible by surgery [8], which allows intravitreal and subretinal administration of vectors to the affected tissue. As retinal cells are differentiated and non-dividing, there is no loss of the transgene even with the use of non-integrating vectors [9]. Finally, there are many different non-invasive approaches that are able to monitor disease progression [10]. Examples include fundus autofluorescence that provides a topographical map of lipofuscin changes in the RPE cells, spectral domain OCT to assess retinal thickness and photoreceptor layer architecture, as well as other known tests like visual acuity and biomicroscopy [11,12,13].

**Figure 1:**
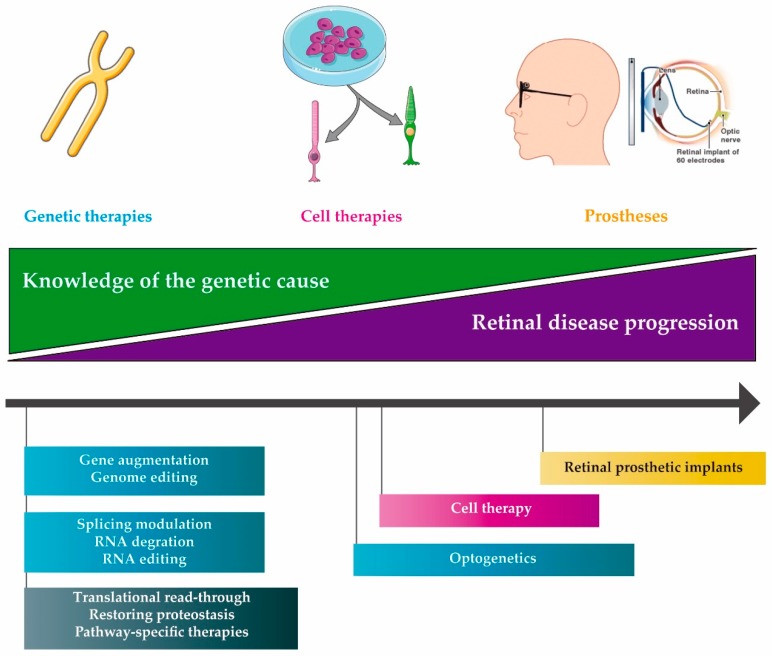
Schematic representation of potential therapeutic approaches according to the retinal disease progression and the knowledge of the genetic cause of the retinal disease. Genetic therapies (blue boxes) are preferred in the first steps of the disease progression (retinal cells are still alive) and when knowledge of the genetic causes of the diseases is present. As the disease progresses and the knowledge of the pathogenesis decreases, other approaches such as cell therapies (pink box) or retinal prosthetic implants (yellow box) can be used. Compound therapies (black box), based on pharmacological treatments, could be used as an alternative approach when the genetic cause of the disease or the pathway involved are either known or unknown. For late-stage diseases, optogenetics or retinal prostheses may be the only option. Image sources—smart.servier.com and Doheny Retina Institute (new.bbc.co.uk/2/hi/science/nature/6368089).

Currently, there are various (overlapping) approaches to treat IRDs under development, including molecular therapies but also stem cell-based therapies and retinal prostheses [14,15,16,17,18] (Figure 1). Despite the promising results obtained with some of these approaches, there are still many challenges that must be overcome in order to reach a broad implementation of treatment modalities for IRDs. The great heterogeneity of these diseases [19] hampers the development of a common treatment for a large number of patients [20]. In addition, a significant proportion of genes involved in IRDs has a cDNA size that exceeds the cargo capacity of adeno-associated viral (AAV) vectors, generally considered the most preferred viral vector for retinal delivery of therapeutic molecules [21]. Finally, the costs of developing gene or even mutation-specific approaches are substantial, while often having only a limited number of individuals that could potentially benefit from a given therapeutic molecule.

In this review, we summarize the current state-of-the-art of the therapeutic approaches for IRDs, with a strong emphasis on molecular therapies.

## 2. Molecular therapies

Due to the severity and heterogeneity of IRDs, avid research is ongoing to identify therapeutic strategies that could ameliorate symptoms and/or disease progression, including many that focus on resolving the consequences of a particular genetic defect [22]. However, a good candidate for molecular therapies requires—(i) a substantial disease burden and a favourable risk/benefit ratio compared to another therapy, if any; (ii) the relevant gene/locus involved in the disease has already been identified and there is ample knowledge of the molecular mechanism of disease and its progression; (iii) the right target cell(s) can be reached, with or without using a therapeutic vehicle; (iv) phenotypic improvement can preferably be achieved with limited expression levels of the therapeutic gene, while its overexpression does not exert any toxic effect [23].

**Figure 2:**
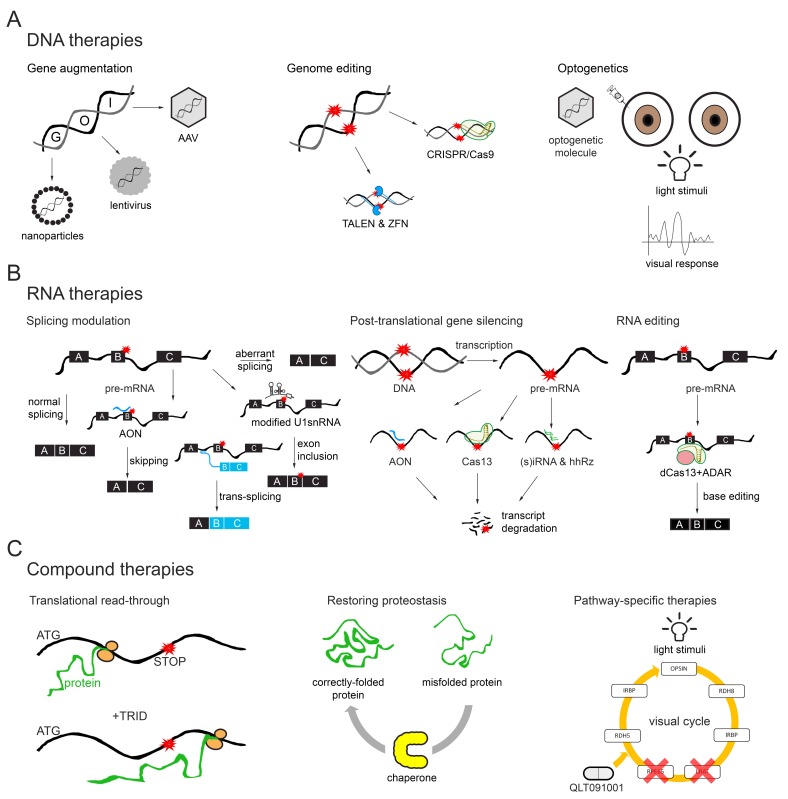
Schematic and simplified representation of the several types of molecular therapies. (**A**) DNA therapies are represented by gene augmentation, genome editing and optogenetics. In gene augmentation, the entire coding sequence of the gene of interest (GOI) is delivered using different vectors. Genome editing employs nucleases able to edit the DNA at a specific position; CRISPR/Cas9 is depicted in green, guide RNA in dark yellow and ZFN and TALEN in blue. Mutations are depicted in red. In optogenetics, a light-sensitive molecule is delivered to the eye to give photosensitive properties to remaining retinal neurons. (**B**) RNA therapies; splicing modulation can be achieved using AONs (in dark blue) for (pseudo)exon skipping or modified U1 snRNA (in black) to favour exon inclusion in cases where mutations (in red) are found in the donor splice site. Trans-splicing occurs between two independent RNA molecules—The original transcript and the exogenous molecule without the mutation. In all cases, splicing is modulated to obtain a transcript with full or residual function. Post-translational gene silencing can be achieved by degrading the RNA transcript using AONs (dark blue), Cas13 (green) with a guide RNA (dark yellow) or (s)iRNA and hhRz (in green). These approaches can be used for dominant-negative mutations by promoting allele-specific degradation. Mutations are indicated in red. With RNA editing using CRISPR/Cas technology, dead Cas13 (in green) is conjugated with an adenosine deaminase (dark yellow) acting at the RNA level (ADAR, in light red). This molecule is guided to the mutation using a guide RNA binding on top of the mutation (in red) to induce a G-to-A transversion. (**C**) Representative examples of compound therapies. Translational read-through allows the ribosome to continue protein synthesis despite a premature stop codon (in red) in the presence of the translational read-through-inducing drugs (TRIDs). Restoring proteostasis can be accomplished by using chaperones to properly fold proteins. QLT091001 is a pathway-specific therapy that acts in the visual cycle and can be used when for example, *RPE65* or *LRAT* are mutated.

We can subdivide the molecular strategies into different groups—DNA, RNA and compound therapies, as graphically depicted in Figure 1 and Figure 2. Ongoing clinical trials for genetic subtypes of IRDs are summarized in Table 1 (DNA therapies), Table 2 (RNA therapies) and Table 3 (Compound therapies).

### 2.1. DNA Therapies

#### 2.1.1. Gene Augmentation

In gene augmentation therapy (also known as gene replacement therapy), a normal copy of the mutated gene is inserted into the host cells using therapeutic vectors (for details on the various types of vectors, see Section 3). To enable this, the gene of interest could be delivered as DNA, as messenger RNA (mRNA) or as mRNA analogue. The big advantage of the mRNA platform is that it does not require delivery into the nucleus and the risk of integration into the host genome is reduced [24,25]. Two of the main challenges of mRNA delivery however are immunogenicity and stability of the RNA molecule [24,26]. Thus, since sustained production of the protein of interest is required for continued improvement of visual function, the DNA platform is the preferred strategy for ocular gene augmentation therapy [25]. With this, the gene of interest is introduced to the cell nucleus, where it often remains in an episomal state while promoters and enhancers can facilitate its expression [25,27].

DNA can be delivered using one of several different vectors but in particular for IRDs, adeno-associated viruses (AAVs) have been most commonly used due to their high tropism for certain retinal cells and their low immunogenicity [14]. Other vectors under study are lentiviruses and nanoparticles, which have larger cargo capacities [28].

Advanced stages of retinal degeneration are not compatible with the use of gene augmentation therapy, since this approach requires the target cells to be alive [29]. Moreover, reaching substantial levels of gene expression is crucial for a significant and strong rescue of the phenotype [30]. This requirement can for instance be improved by varying the serotypes of AAVs that are used to deliver the transgene [31,32,33], by varying the promoter sequence [30] or by producing codon-optimized cDNA versions of a given gene [34,35].

For IRDs, gene augmentation is currently the most advanced therapeutic strategy, with market approval for one gene (*RPE65*, Luxturna^TM^), several ongoing clinical studies for other genes (Table 1) and preclinical development for a plethora of genes mutated in these diseases [36]. Despite these promising results, gene augmentation strategies may not be the best approach when treating dominant conditions [37], as the mutated allele underlying the disease most often first needs to be inactivated such that it does not interfere with the normal allele [38]. Such an allele-specific inhibition of expression can be achieved either at the DNA level (Section 2.1.2) or at the RNA level (Section 2.2) and, if needed, can be combined with gene augmentation [39]. In addition, the cargo capacity of AAV remains a challenge, since several of the recurrently mutated genes (e.g., *USH2A, EYS,* etc.) are by far exceeding it. Several studies using dual or even triple AAVs [40,41,42,43], or microgenes (smaller versions of the gene) [44] are ongoing.

#### 2.1.2. Genome Editing

In addition to gene augmentation, precise editing of genomic DNA has gained an enormous attention over the last five years. Currently, there are two main approaches in genome editing—an in vivo approach, in which the disease-causing mutations are corrected inside the retina and an *ex vivo* approach to first correct the mutation in patient-derived cells, which can be followed by cell transplantation [45]. The editing itself can be achieved by different classes of molecules, as outlined below.

**ZFNs and TALENs:** Zinc-finger nucleases (ZFNs) and transcription activator like effector nucleases (TALENs) are molecules that pioneered genome editing. Both can induce a wide range of genetic modifications by generating double-strand breaks (DSBs) in the DNA that subsequently stimulate one of the DNA repair pathways of the cell—non-homologous end-joining (NHEJ), homology-directed repair (HDR) or microhomology-mediated end joining [46,47]. To induce DSBs, either nuclease needs to be guided to its target sequence by a DNA-binding protein domain. These approaches are thus depending on the engineering of new proteins for each target, making them laborious and challenging [48]. Nevertheless, ZFNs have been used as a proof-of-concept treatment in IRD. Human embryonic cells carrying the c.68C>A; p.P23H mutation in *RHO* were targeted using ZFNs, which led to an increase in homologous recombination events when the ZFNs were transfected with a homologous donor template, compared to delivery in the absence of this template [49].

TALENs have been used in the correction of the *Crbrd8* allele in C57BL/6N mice. HDR triggered by a combination of TALEN and a single-stranded donor oligonucleotide repair template was observed in 27% of the treated mouse embryos and resulted in an improvement of retinal function [50].

**CRISPR/Cas system:** The CRISPR/Cas system is considered a more advanced genome editing tool compared to ZFNs and TALENs, as it presents many advantages including the simple design of the target, its higher efficacy and its ability to introduce mutations in multiple genes at the same time by using a combination of guide RNAs (gRNAs) [25]. The CRISPR/Cas system has been improved to a simpler version, CRISPR/Cas9, which nowadays is commonly used for mammalian genome editing [51,52,53]. The Cas9 endonuclease is delivered into a cell in conjunction with a gRNA, after which it can cut the genome specifically at any desired location [52,54].

As for other genome-editing tools, its major limitation is the possibility of off-target effects [46]. To overcome this aspect, researchers have developed different strategies—(i) using ribonucleoproteins (RNPs) as a form of delivery [55]; (ii) titrating the concentration of gRNA and Cas9 or using two gRNAs flanking the target region [56]; (iii) adding two additional guanine bases at the 5’ end of the gRNA sequence for Cas9 derived from *Streptococcus pyogenes* (SpCas9); interestingly, this modification in the design did not reduce the on-target ratio both in vivo and *in vitro* [57]; (iv) selecting unique DNA target sites with no homology to any other gene sequence [58]; (v) using paired Cas9 nickases, modified Cas9 nucleases that cut only one strand of DNA and thus can produce DSBs with two times more specificity [52,57,59], (vi) employing a high-fidelity version of Cas9 (SpCas9-HF1) [59] or (vii) using the ‘enhanced specificity’ SpCas9, which is reported to not only decrease the off-target events but also to obtain a higher on-target efficacy [60]. All these strategies have allowed researchers to yield relatively high editing rates *in vitro* but these rates are generally lower in vivo, for instance in treated retinas [52,61,62,63,64,65,66,67,68,69,70,71,72]. The first challenge is the delivery of the CRISPR system directly into the cells or tissue of interest. As it was previously mentioned, AAV vectors are currently considered the most potent therapeutic vector for retinal delivery. However, its limited cargo capacity hampers an efficient delivery of the complete CRISPR/Cas9 system [73]. One solution for this problem is the delivery of the SpCas9 and gRNA separated into two vectors, with which Hung and colleagues obtained an editing efficacy of 84% in the mouse retina [52]. Other studies using two AAV vectors have also obtained good results over the last years, although it should be mentioned that these in vivo studies only used CRISPR/Cas9 technology to activate NHEJ, resulting in indels and subsequent gene inactivation [55,74,75,76,77]. A major concern that remains to be solved is how to increase the efficacy of genome correction in the retina, since photoreceptors are post-mitotic cells that to a large extent lack HDR mechanisms. Suzuki and colleagues developed a strategy coined homology-independent targeted integration which allows for targeted knock-in in non-dividing cells like photoreceptors [45,77]. This appears as a promising approach, as it is based on the NHEJ mechanism, as opposed to HDR, for specific integration of a desired DNA sequence [45,77].

Besides precise editing, the CRISPR/Cas9-system can also be used in other ways, such as the invalidation of mutant alleles that underlie autosomal dominant IRD (reviewed by Diakatou et al.) [78], or the removal of intronic sequences harbouring pathogenic pseudoexons. For the latter, Maeder and colleagues recently developed a CRISPR/Cas9-based approach (EDIT-101) for the splice mutation c.2991+1655A>G in *CEP290*. EDIT-101 promotes the deletion of part of intron 26 where the deep-intronic variant is located. It was tested in a humanized *Cep290* mouse model carrying the c.2991+1655A>G variant [79] and a comparable surrogate non-human primate (NHP) vector also showed efficient editing in photoreceptor and somatic primate cells [80].

Although several hurdles still need to be overcome, the development of the CRISPR/Cas9 system has opened new avenues for the future treatment of various genetic subtypes of IRD.

#### 2.1.3. Optogenetics

Although gene-specific augmentation therapy is a very attractive approach in patients with a known genotype and at a relative early stage of the disease, it is not suitable for patients who present a more advanced stage of the disease in which many of the photoreceptors have been lost (Figure 1).

Optogenetics is a technique used to monitor or control neural activity with light, which can be achieved by the genetic introduction of light-sensitive proteins into retinal cells. This strategy is often used to convert secondary or tertiary neurons into “photoreceptors” or, less often, to restore sensitivity of degenerating photoreceptor cells [81,82,83]. One advantage of optogenetics is that it may more precisely excite the neural pathway compared to for example, electronic retinal implants [82,84]. In addition, optogenetics can be used independent of the primary genetic defect.

Opsins represent the major optogenetic tool [85]. They function as sensory receptors or light-responsive ion channels. Two types of opsin have been described—microbial opsins (type I) and animal opsins (type II) [81,82]. Type-I opsins (channelrhodopsins, halorhodopsins and archaerhodopsins), following light capture, result in a passive flow of ions across the cell membrane. When they are introduced into non-light sensitive cells, microbial opsins can induce rapid optical control of specific cellular processes. Type-I opsins allow high-speed neural activation and silencing, without requiring any additional chemicals [86]. Type-II opsins (rhodopsin and melanopsin) are part of the large family of naturally occurring light-sensitive G protein-coupled receptors (GPCRs). In contrast to microbial opsins, animal opsins present a much higher light sensitivity, as the light signal is amplified by G-protein-coupled signalling cascades [87]. Both types of opsins are small enough to be encapsidated in AAVs [81,82].

Experiments in animal models (mouse, rat and dog) revealed that expression of type-II opsins resulted in increased light sensitivity, within 1–2 log units of the threshold for normal cone vision, although this sensitivity may come at the cost of slower kinetics [88]. In contrast, the use of type-I opsins is superior in terms of kinetics, yet its sensitivity is lower. Both types however are limited in their operational range of light levels and will likely require modification of the incoming light signal [89]. Moreover, many questions still need to be addressed such as the identification of the best vector and surgical approach for delivery. Although a wide range of ubiquitous, photoreceptor-, bipolar- or ganglion-cell specific promoters have been used, the ideal condition so far remains unclear [82].

Currently, there are two clinical trials ongoing that use optogenetics for vision restoration. One comprises a phase I/II clinical trial (NCT02556736; Table 1) and a second trial is still in a recruiting state (NCT03326336; Table 1), without any results reported yet for both trials.

### 2.2. RNA Therapies

#### 2.2.1. Splicing Modulation

**Antisense oligonucleotides (AONs):** Around 15% of all IRD-causing mutations affect the splicing process [90]. Currently, the most widely used genetic therapy to correct aberrant splicing employs antisense oligonucleotides (AONs) [91], small DNA or RNA molecules that bind to their target pre-mRNA in a complementary way [91,92]. Their ability to modulate splicing in fact offers several advantages over gene replacement approaches, especially for IRDs [93]. Initially, AONs were simple antisense RNA molecules but found to be rapidly degraded by nucleases [91]. Therefore, multiple chemical modifications for the backbone or sugar groups were added to improve their target affinity and resistance to nuclease activity [91,94]. These new AONs were classified as first or second generation AONs, depending on their chemical modifications [94]. A third generation class of AONs consists of analogues of nucleic acids. Due to their relatively small size, it has been shown that AONs can be delivered to the eye either as naked molecules or in an AAV using a modified U7-snRNA system [95,96].

The first approved AON-based drug was Formivirsen, also known as Vitravene [97]. Vitravene was used to treat cytomegalovirus retinitis in patients whose immune system was compromised [97,98]. In the last years, the number of IRD-causing mutations that affect pre-mRNA splicing has rapidly increased. An example is a recurrent deep-intronic mutation in *CEP290* (c.2991+1665A>G) underlying congenital blindness [99]. For this mutation, the potential of AON-based therapy was demonstrated first in *in vitro* and in vivo models [96,100,101,102,103,104] and later on, in a phase I/II clinical trial [105]. Promising proof-of-concept studies employing AONs have also been developed for other mutations in *CEP290* [106], and for other genes mutated in IRD such as *OPA1* [107], *CHM* [108], *USH2A* [109] and *ABCA4* [110,111,112,113]. 

Besides the clinical trial for *CEP290* (NCT03140969) there is also an ongoing clinical trial for *USH2A* (Table 2). The results obtained so far indicate that AON-based therapy is a promising therapeutic strategy, albeit only for a selected group of IRD-causing mutations. However, the use of whole genome sequencing or targeted full-gene sequencing will likely identify more mutations amenable for this type of therapy in the near future. Overall, the remaining challenges for AON-based splicing modulation are mainly related to delivery, longevity and potential off-target effects.

**U1 spliceosomal RNA:** Many exonic splice donor site (SD) mutations have been identified in the last nucleotide of an exon and over 95% of these are predicted to result in aberrant splicing [114]. The splicing process needs the recognition of splice sites and subsequent assembly of the spliceosome. The latter is started by formation of stable complexes consisting of U1 small nuclear RNA (U1 snRNA), pre-mRNA and splice factor proteins. Splice donor sites are recognized directly by the U1 complex and are crucial for a proper splicing of exons. In case nucleotides within the splice donor site of an exon are mutated, an attractive therapeutic approach is to use a modified U1 snRNA. Tanner and colleagues demonstrated that a mutation that induces exon skipping in *RHO* (c.936G>A; p.Q311Q) can be rescued by adapting the U1 snRNA to the mutation [114]. In the last years, this approach has also been successfully employed *in vitro*, to rescue a mutation in exon 5 of the *BBS1* gene that underlies Bardet-Biedl syndrome [115], or a mutation in intron 10 causing *RPGR*-associated X-linked RP [116]. Thus, the U1 snRNA system definitely holds some therapeutic potential for mutations affecting the splice donor site. However, possible off-target effects caused by the delivery of an exogenous modified U1 snRNA are still poorly studied [117,118].

**Trans-splicing:** Trans-splicing is a naturally occurring process that results in an alternative processing of the pre-mRNA and was first reported in plants and bacteria [119,120]. Unlike in cis-splicing, trans-splicing takes place between two independent RNA molecules [93,119,120]. In vertebrates, these trans-splicing events occur in some key physiological processes, for example, the regulation of gene expression [121,122,123]. In humans, trans-splicing has also been reported in some diseases such as cancer [120]. 

Therapeutic trans-splicing offers an intriguing strategy to remove mutations from the mRNA. Only the introduction of an exogenous RNA molecule can be sufficient to activate the trans-splicing process. This exogenous RNA, also called PTM (pre-mRNA trans-splicing molecule), is constituted by a binding domain that specifically targets the molecule towards a specific region within the endogenous pre-mRNA, an artificial intron sequence harbouring all the elements required for splicing and the sequence that needs to be replaced [93,119]. For a 5′ PTM, the partial coding sequence has to end at the 3′ end of an exon to allow for a 5′ splice site and vice versa for a 3′ splice site [124]. In the last years, trans-splicing has revealed promising results for the correction of mutations in *RHO* and *CEP290*. In the first example, Berger and collaborators were able to correct *RHO* mutations located in exons 2 through 5 by delivering the correct sequence of those exons in an AAV. This led to successful trans-splicing events, both in vivo and *in vitro* [119]. Dooley and colleagues delivered a part of the *CEP290* gene in an AAV and thereby could successfully replace the aforementioned c.2991+1655A>G mutation using the trans-splicing approach [124]. These data support the usefulness of trans-splicing as a therapeutic tool in IRDs.

#### 2.2.2. Post-transcriptional Gene Silencing

**iRNA and Ribozymes:** Both Hammerhead ribozymes (hhRz) and (short) interference RNA ((s)iRNA) catalyse the sequence-specific cleavage of target mRNAs. iRNA molecules are double-stranded RNAs that are able to inhibit gene expression by binding to specific mRNAs (cellular or viral) [93,125]. Despite their affordability and speed, the effect of iRNA is often incomplete and temporary, with potential off-target effects [126]. In addition, variations between experiments and laboratories often occur. These variations limit the broad application of iRNA technology in many diseases including IRDs [46], although promising results have been obtained in age-related macular degeneration, a multifactorial subtype of retinal disease. Specifically, Ryoo and colleagues used a novel siRNA-based anti-VEGF nanoball that, upon intravitreal administration in mice, showed therapeutic effects for at least two weeks [127].

HhRzs are small RNA molecules causing enzymatic cleavage of polyribonucleotides [128]. The hhRz consists of three helixes surrounding an evolutionary conserved catalytic core. This gives rise to an antisense complementary region that provides the unimolecular RNA the capacity to recognize and subsequently enzymatically cleave its target mRNA [128].

Both types of molecules (iRNA and hhRz) have been successfully used to degrade an incorrect *RHO* transcript responsible for dominant retinitis pigmentosa, a common subtype of IRD [128]. Another alternative is employing microRNAs (miRNAs), which act at the post-transcriptional level to regulate gene expression in the retina. miRNAs generally bind to mRNA and cause a reduction of translated products. Some miRNAs are commonly expressed in all retinal cell types while others are specifically expressed in one or the other [5,129], suggesting that there are possibilities to employ these molecules therapeutically. However, more studies are needed before miRNAs can actually be used [130]. Overall, post-transcriptional gene silencing mediated by iRNA and ribozymes are believed to be a promising strategy for treating dominant-negative mutations.

**RNAse H-dependent AONs:** Besides redirecting splicing, AONs can also be used to specifically degrade transcripts, even in an allele-specific manner. Some oligonucleotide modifications combine AON segments with conformationally restricted residues that affect cleavage of their intended targets [131]. With this, catalytic activation of RNase H, a ubiquitous enzyme cleaving the RNA part of DNA/RNA hybrid duplexes, can be induced. The big advantage of these hybrid duplexes is that only a low amount of AON is sufficient to induce catalytic turnover. Furthermore, this catalytic turnover provides enough time for AONs to act as a potential drug due to their stability in blood serum, that is, a few days [132]. Murray and colleagues used rodent models genetically modified for *RHO* (p.P23H) to test RNAse H-activating AONs in vivo. They observed that the AON-mediated knockdown of mutant p.P23H rhodopsin expression reduced photoreceptor degradation, thereby preserving the function of photoreceptors in the transgenic rats [133]. Recently, AON-mediated transcript downregulation has also been assessed *in vitro* for a *NR2E3* variant underlying autosomal dominant RP [134], underscoring the usefulness of this approach for some (dominant) mutations.

**Cas13:** The editing system based on Cas13 can reduce off-target effect rates shown by other systems as explained previously [135]. RNA-guided RNA-targeting CRISPR-Cas effector Cas13 (previously named C2c2) can be engineered to bind and subsequently knockdown mammalian RNA [136]. Abudayyeh and colleagues verified that, for endogenous genes, the knockdown efficiency is transcript-dependent. Despite, the efficacy was comparable to that shown by iRNA, thus the substantially lower off-target ratio makes this approach well-suited for therapeutic applications [137].

#### 2.2.3. RNA Editing (dCas13 and ADAR)

The ADAR (adenosine deaminase acting on RNA) family of proteins can mediate endogenous editing of transcripts via the deamination of adenosine to inosine, a nucleobase that is functionally equivalent to guanosine both in splicing and in translation [135]. Cox and colleagues designed a catalytically inactivated Cas13 (dCas13) that is able to retain its RNA-binding capacity, to direct an ADAR towards the RNA transcript of interest and to perform its adenosine-to-inosine deaminase function [135]. This demonstrated the flexibility of Cas13 to be adapted as a tool for nucleic acid modification. The system that was created is called REPAIRv2 and generates a higher specificity compared to other RNA-editing platforms reported so far [138,139] with high levels of on-target activity. Other advantages include—(i) Cas13 has no targeting sequence constraints and does not present any preferential motif surrounding the target adenosine, allowing any adenosine in the transcriptome to be potentially targeted; (ii) the REPAIRv2 system directly deaminates target adenosines to inosines and does not depend on endogenous repair pathways, thereby enabling RNA editing in post-mitotic cells like neurons and photoreceptors and, (iii) RNA editing, contrary to DNA editing, is transient and thus can be more readily reversed, allowing temporal control over editing events [135]. These features make RNA editing an interesting strategy to be used in future therapeutic studies in IRDs. 

### 2.3. Compound Therapies

As happens in other types of diseases, pharmacological development can offer an entirely different approach to the treatment of IRD. However, the great heterogeneity observed in IRDs and the access-limiting BRB present major challenges towards an effective compound therapy [140]. In this section, we focus our attention on some promising drugs for the treatment of IRDs.

#### 2.3.1. Translational Read-Through

Translational read-through (TR) therapy is based on small molecules, also known as TR-inducing drugs (TRIDs), that allow the translation machinery to bypass a premature termination codon (PTC) during translation [141]. In addition, PTCs can induce mRNA degradation through nonsense-mediated decay and thereby also inhibit full-length protein expression [141,142]. The incorporation of an amino acid at the site of the premature stop codon can increase the expression of the full-length protein as well as the reduction of nonsense-mediated decay [142]. Until now, the detailed mechanisms by which TRIDs induce their therapeutic effect are not completely understood. However, it is known that TR efficiency depends on the competition between decoding of the stop codon by a near-cognate tRNA and stop codon recognition by eRF1 [141]. There are two main classes of TRIDs—aminoglycoside and non-aminoglycoside TRIDs. From the first group, gentamicin has been most widely used to analyse TR in different disease models, including those affected with IRD. The efficacy of gentamicin was studied in different rat and mouse models—(i) the S334ter rat model that carries a nonsense mutation (c.1002T>A; p.S334*) in the gene encoding the visual pigment rhodopsin (*Rho*) and (ii) the *rd12* mouse, a model for retinal degeneration caused by a nonsense mutation in *Rpe65*. Systematic gentamicin treatment showed different results between the two models. In S334ter rats, a partial rescue of photoreceptor survival was noticed however, no rescue was observed in *rd12* mice [143]. Studies on genes mutated in Usher syndrome (a syndromic form of IRD accompanied by hearing impairment) demonstrated that aminoglycosides and derivatives thereof can mediate TR of different disease-causing PTCs in the *PCDH15* and *USH1C* genes, in *in vitro* translation assays as well as in cell culture experiments [144,145,146,147,148,149].

The non-aminoglycoside TRID PTC124 (also known as Ataluren) is used in a wide range of diseases including Duchenne muscular dystrophy and cystic fibrosis [150,151,152,153], with several clinical studies having been performed or ongoing. For IRDs, promising results using PTC124 have been reported by the restoration of full-length RP2 protein, the encoding gene of which is mutated in X-linked RP, as well as for the REP1 protein, encoded by the *CHM* gene that is mutated in choroideremia. In X-linked RP, rod photoreceptors mainly suffer from the loss of RP2, although the effect on cone photoreceptors and RPE cells should not be neglected. Schwarz and colleagues used TRIDs (both the aminoglycoside G418 as well as PTC124) to successfully increase full-length RP2 protein levels in the presence of the p.R120ter (c.358C>T; p.R120 *) mutation. In choroideremia, many nonsense mutations in *CHM* have been described. Several studies attempted to use TRIDs, including PTC124 or its analogue PTC-414, to increase REP1 protein levels, either in cellular (human fibroblasts, iPSC-derived RPE) or animal (zebrafish) models [154,155,156]. The efficacy of read-through was found to be considerably variable, not only depending on the type of nonsense codon and its surrounding sequence but also on the remaining transcript levels that can differ significantly between patients [142]. Another study, based on topical administration of PTC124 to the eye, demonstrated functional restoration of the harmonin protein in a mouse model for Usher syndrome type 1c (USH1C) [141,145].

One of the disadvantages of the systemic use of TRIDs is that for many diseases, drugs need to go through physical barriers (such as the blood-brain barrier or BRB). These barriers can reduce compound availability in the targeted organ after treatment, illustrating the need to increment the dosage administered to the patients or to change the delivery method of TRIDs (commonly intraperitoneal) towards local administration [141]. One strategy to overcome this is the encapsulation of drugs into tissue-specific liposomes [157]. In particular for IRDs, topical applications or intraocular injections should be considered in the future. 

#### 2.3.2. Restoring Proteostasis (Protein Therapies) 

Photoreceptor cells require a rigorous regulation of proteostasis (maintaining a healthy protein balance) to ensure their correct function and viability. As a result, certain genetic changes in specific genes with an essential function in the photoreceptor cell can have dramatic effects. One example is the dominant-negative effect of mutations in *RHO*, which leads to the disruption of outer segments in the photoreceptor cell [158]. The manipulation of proteostasis mechanisms such as the heat-shock response (HSR) or unfolded protein response could be a good therapeutic target in order to alleviate misfolding diseases (e.g., targeted up-regulation of these pathways to reduce aggregation of proteins such as rhodopsin) [159]. This could be achieved either by the administration of drugs that restore proteostasis (e.g., pharmacological chaperones, kosmotropes, molecular chaperones or autophagy inducers) or by targeting key molecules in the photoreceptor proteostasis network [160,161].

So far, “proteostasis” therapies are mainly focused on rhodopsin, as *RHO* is one of the most frequently mutated genes in autosomal dominant RP. One of the most common mutations in this gene is the p.P23H mutation, which acts in a dominant-negative fashion on wild-type rhodopsin by inducing misfolding [160]. Intriguingly, it was reported that misfolded proteins could be rescued by a ligand that works as a chaperone [161].

Pharmacological chaperones are small, substrate-specific molecules that are able to directly target the protein structure and shift the protein folding equilibrium towards its native state [158,162]. Studies using this kind of chaperones suggested that correction of mutant rhodopsin folding, without improving its stability, could further compromise the outer segments and increase rod cell death. Thus, these data underscore the necessity to ensure opsin stability using parallel treatment strategies [163,164].

Kosmotropes are small, low molecular weight compounds that can enhance the stability of proteins in their native conformation and decrease aggregation. Kosmotropes bind to proteins non-specifically and thus have the potential to be used in a wide range of protein-misfolding diseases [161]. The chemical chaperone tauroursodeoxycholic acid (TUDCA) has been studied in several retinal degeneration models [165,166,167], although its efficacy was found to vary between different studies, even in the presence of the same IRD-causing mutation, namely p.P23H, in the gene encoding rhodopsin. It has been hypothesized that activation of PERK, one of the receptors responsible for UPR [168] differs between models, thereby explaining the ineffectiveness of TUDCA in some of them [161,165].

Finally, another alternative to restore proteostasis is through the control of the adaptative machinery that is responsible for its maintenance via HSR and UPR [158]. Inhibition of molecular chaperones can activate the HSR by releasing HSF1 and inducing cell stress, which triggers the production of molecular chaperones. The Hsp90 inhibitors and HSR inducers geldanamycin, radicicol and 17-AAG reduced P23H aggregation and cell death *in vitro* [162]. Hsp90 works as a crucial chaperone in the maturation of many proteins. Targeting Hsp90 directly however could affect normal vision, suggesting that an alternative therapeutic approach could include the stimulation of both the HSR and UPR pathways with arimoclomol (a heat shock protein inducer). In rat models carrying the p.P23H rhodopsin mutation, it has been demonstrated that administering arimoclonol via intraperitoneal injection reduced mutant rhodopsin aggregation and ameliorated photoreceptor degeneration [160,161].

#### 2.3.3. Pathway-Specific Therapies 

Cyclic guanosine-monophosphate (cGMP) is a crucial molecule for photoreceptor signal transduction and has two main cellular effectors—cyclic nucleotide gated ion channels (CNGCs) and cGMP-dependent protein kinase G (PKG) [169]. It has been reported that over-activation of PKG can be enough to cause photoreceptor cell death and that its activation levels are higher in mutant photoreceptors. Knowing that CNGCs play an important role in driving phototransduction, some interesting observations have been made. The deletion of CNGC beta subunits protects photoreceptors in *rd1* mice that harbour a defect in the *Pde6b* gene, which encodes the phosphodiesterase protein involved in the phototransduction cascade. Therefore, either PKG or CNGCs can be considered as critical disease drivers; consequently, both of them are therapeutic targets for prevention of (further) retinal degeneration [140]. As mentioned above, the BRB can prevent the access of therapeutic agents to the retina. Vighi and colleagues overcame this problem using a liposomal drug delivery method, the liposomal cGMP analogue formulation LP-CN03, and demonstrated improved visual function as well as reduced photoreceptor degeneration in mouse models harbouring mutations in different IRD gene orthologues [140]. Together, these data suggest that cGMP signalling could be a common pathway to target for the treatment of genetically and phenotypically divergent kinds of retinal degeneration [140,169].

Another drug previously tested in clinical trials is an orally delivered synthetic cis-retinoid also known as QLT091001. This drug triggered visual restoration in transgenic and naturally occurring mouse and dog models mutant for *Rpe65* [170]. The results of the clinical trial NCT01014052 (Table 3) showed that QLT091001 did improve visual function in subjects with IRD due to *LRAT* or *RPE65* mutations. Mutations in these genes can cause different subtypes of IRD, usually classified as LCA or RP. Despite this, the exact genetic defect apparently did not affect the drug response in both LCA and RP patients [171]. Upon oral administration of QLT091001, patients showed beneficial effects in the remaining photoreceptors of the retina in both eyes, although it did not stop the progression of photoreceptor degradation completely. The safety profile of this drug showed transient adverse effects such as headaches or nausea [171]. In theory, QLT091001 could be combined with gene augmentation therapy, although the combination of treatments, in particular for orphan diseases, poses additional challenges on drug development.

## 3. Delivery of Therapeutic Molecules

### 3.1. Methods of Ocular Delivery

Despite its small size, the eye contains several cell and tissue types that can be targeted by therapeutic agents [172]. Local tolerability of vector administration in IRDs has been reported in various studies and no serious systemic problems have been indicated so far [23]. Commonly, we can identify two main potential delivery methods—subretinal and intravitreal injections. Subretinal injections are considered to be more prone towards complications (e.g., retinal detachment), especially in patients with affected retinal integrity, compared to intravitreal injection. Despite, the vector is delivered much closer to its target cells/region, allowing an efficient vector transduction in RPE cells and/or photoreceptors [173]. In contrast, intravitreal injections allow an easier targeting of the optic nerve, lens or inner retina and less often the outer retina or the anterior chamber. It also shows fewer procedure-related complications but the transduction of the viral vector into photoreceptors and RPE cells is less efficient when compared with subretinal injections [23,174]. Apart from these two methods, there is an alternative system—Suprachoroidal delivery. With this, therapeutic agents are delivered directly to the suprachoroidal space located between the sclera and choroid [175,176]. Preclinical animal studies showed that suprachoroidal drug delivery has the same safety profile as intravitreal injections [177,178,179]. Results from completed clinical trials (e.g., NCT01789320) have also showed encouraging safety profiles [180], although potential spreading of therapeutic vectors into the systemic circulation needs to be considered.

Finally, there is the possibility of topical delivery by the use of eye drops. However, this approach can result in lower bioavailability and increased clearance in comparison to the different types of injections. In addition, ocular barriers decrease the bioavailability of topically applied therapeutic agents to less than 5% [181]. Therefore, the effectiveness of this method is less than those reported with the systems previously described.

### 3.2. Vectors

Besides the routes of administration, a wide range of viral and non-viral gene delivery approaches has been developed over the last twenty years, in order to allow an efficient transfer of therapeutic molecules to the right target cell. Choosing one over another depends on the cell or tissue type to be targeted, the cloning capacity of each of the vectors and safety concerns [23].

#### 3.2.1. Viral Vectors

**Adeno-associated viruses (AAVs):** Most commonly but not exclusively, therapeutic genes are delivered to their target cells by viral vectors. AAVs are the most common viral vectors used for gene therapy in IRDs, mainly due to their low immunogenicity and toxicity, and allow for long-term transgene expression [182,183].

Recombinant AAV (rAAV) is the most popular viral vector for delivery in IRDs. Its tropism for different types of retinal cells allows efficient transduction of these cells and relatively fast and stable transgene expression [184]. rAAV is available in two forms, single-stranded AAV (ssAAV) and self-complimentary AAV (scAAV). Once a cell is transduced with ssAAV, the single-stranded DNA of the virus needs to be converted into the double-stranded form; this is a rate-limiting step that can be circumvented with scAAVs [25]. The scAAVs are engineered in a way that upon infection two complementary halves will generate a double-stranded DNA, promoting faster and increased transgene expression [185]. However, the main disadvantage of scAAVs is their reduced cargo capacity (2.4 kb), compared to ssAAV (4.8 kb) which limits its transgene selection. scAAV vectors have been demonstrated to transduce retinal ganglion cells, photoreceptors and RPE cells, and can lead to transgene expression within two days after injection in mice while ssAAV requires several weeks [186]; this rapid and increased transgene expression was independent of the AAV serotype tested (i.e. -1, -2 or -5) [25].

Despite its numerous advantages, AAV presents a limited packaging capacity (4.8 kb) [22,25,45]. To overcome this, various strategies have been developed. For instance, with the use of dual AAV vectors, a cDNA can be split in two separate fragments, after which the two parts recombine inside the cells using HDR, trans-splicing or both [41,187]. With this, the capacity can be increased up to 9 kb [41,43,187]. The dual AAV system has been used in preclinical studies to deliver some genes mutated in IRD such as *ABCA4* [188] and *MYO7A* [189], with promising results. However, dual AAVs may still not be sufficient for several other genes mutated in IRD. The use of triple AAV approaches allows to increase the cargo capacity to 14 kb [43,189,190], enabling the development of gene therapies for IRDs caused by mutations in even larger genes. Maddalena and colleagues demonstrated the potential of this system to restore *CDH23* (cDNA size 11.1 kB, mutated in Usher syndrome type 1D) and *ALMS1* (cDNA size 12.9 kb, mutated in Almstrom syndrome) gene expression. Certainly, the enclosed and small subretinal space may facilitate co-infection of the same cell by three independent AAV vectors [43].

To date, up to 12 AAV serotypes and more than 100 variants have been identified in humans and NHPs [25]. From these, AAV2, AAV5 and AAV8 have been most extensively studied, being used as delivery vector in several clinical and preclinical studies for different genes such as *RPE65, CNGB3, RS1* or *PDE6B* (Table 1) [25]. AAV serotypes 2, 5, 7, 8 and 9 are able to transduce photoreceptors, while virtually every AAV serotype is capable of efficiently infecting RPE cells [23]. So far, AAV2 is the only vector described to transduce retinal ganglion cells upon intravitreal delivery [191,192]. However, alternative serotypes of AAV have also been developed. Some examples are the serotypes AAV7m8 [193] and AAV8BP2 [194]. Both of them were designed to alter AAV capsids aiming to improve uptake into the retina and be able to target photoreceptors following intravitreal injection [193,194]. AAV7m8 serotype was generated by in vivo directed evolution in the mouse retina by Dalkara and collaborators [193,195]. They reported that the intravitreal delivery of AAV7m8 resulted in effective pan-retinal photoreceptor and RPE cell transduction in mice. This serotype was also tested in NHP retinas via intravitreal administration. The results indicated a higher transduction rate of foveal and extrafoveal photoreceptors in comparison with the parental serotype AAV2. However, authors also noted toxicity in an NHP injected with a high titre of AAV7m8 [193]. Therefore, a study of safety and efficiency of transduction using lower doses of this serotype is required if this capsid is planned to be used in primates, including humans. The AAV8BP2 serotype was generated in vivo by Cronin and colleagues, it targets ON-bipolar cells and cone photoreceptors efficiently following both intravitreal and subretinal injections in mouse [194]. This research group performed a comparative study between these two novel serotypes. Both vectors showed promising results in mice but in NHPs, either subretinal or intravitreal delivery did not allow to transduce all target cells within the retina. Specifically, AAV7m8 showed some toxicity effects (as previously reported) as well as severe inflammation when using a high titre [195]. For AAV8BP2, a lower transduction of bipolar cells was observed, even at high doses. Taken together, the results of both serotypes indicate that studies in rodents might not provide sufficient information to understand the cellular transduction and pharmacological properties of these engineered AAVs and further studies in for example NHPs are needed before using them in humans. Carvalho and collaborators characterized an *in silico* designed serotype named Anc80 for retinal gene transfer [196]. Three Anc80 variants were evaluated, Anc80L27, Anc80L65 and Anc80L12, in mice and NHP. All of them were capable of efficiently targeting retinal cells following subretinal delivery, although Anc80L65 showed a higher efficiency for targeting retinal cells as well as higher expression levels compared to AAV8. These data support the use of Anc80L65 for gene delivery to the retina. Taken together, all these studies characterizing novel serotypes have illustrated the impact of minimal changes in capsid composition on aspects relevant to experimental and clinical gene transfer applications. This will prove useful for improving delivery to the retina and thus for their use in developing new treatments for IRDs.

**Lentiviral vectors:** Lentiviruses (LV) are RNA viruses of the retrovirus family. In IRDs, the retroviral variant of human immunodeficiency virus type 1 (HIV-1) or the equine infectious anaemia virus (EIAV) have been tested. LVs have the ability to pass through the cells’ intact nuclear membrane and infect both dividing and non-dividing cells [197]. Moreover, LVs efficiently integrate their genome into that of the host-cell, leading to stable expression. LVs are modified to stop replication, so these vectors are not pathogenic after initial gene delivery. LVs present a transgene capacity cargo of ~8–10 kb and, in terms of IRD, are capable of infecting RPE cells and to a lesser extent of differentiated photoreceptors [23,25]. EIAVs have been used for *ABCA4*, as its cDNA size (6.8 kb) outplaced the cargo capacity of AAV but not that of lentiviruses [198]. To assess this strategy in human subjects, a clinical trial (NCT01367444) has been ongoing for several years (Table 1), whilst no clear efficacy data have been reported.

**Adenoviruses and helper-dependent adenoviruses:** In comparison to the previously mentioned viral vectors, adenoviruses (AdVs) have a cargo capacity of about 8 kb, while its ‘gutted’ helper-dependent adenovirus (HDAd) has an extremely large capacity of up to 36 kb. It is demonstrated that adenoviral vectors infect RPE cells efficiently but because the coxsackie-AdV receptor is presumed to be absent on the cell membrane of photoreceptors [199,200], these vectors do not efficiently transduce photoreceptor cells [201]. Overall, AdVs have been only sparsely used in the treatment of IRDs [202,203].

#### 3.2.2. Non-Viral Vectors

Although various viral vectors have demonstrated their potential in the treatment of IRDs, there is a continuing need for refinement delivery vectors for the eye. Therefore, research efforts have also been directed towards the development of non-viral delivery systems, such as nanoparticles (NPs), naked DNA or liposomes [174].

Naked DNA is the most elementary form of non-viral gene therapy [204]. As naked DNA does typically not enter into the cells, it is not considered as a suitable therapy for the eye. Different studies have used electroporation or iontophoresis to obtain a significant uptake and expression in the target cells, however both delivery ways presented significant challenges. The side effects of electroporation make it an unlikely method to be clinically feasible, whilst iontophoresis so far presents conflicting information about the real effectiveness of this method [174,204].

In contrast, NPs are well capable of delivering plasmid DNA containing a functional copy of a gene into the retina [205]. The three determinants of the effectiveness of gene delivery using this kind of vectors are cellular uptake, endosomal escape and transfer of the plasmid DNA into the nucleus. All forms of NPs-(metal, lipid or polymers) have some capability to pass through the cell membrane, avoid endosomal trapping and deliver the plasmid DNA into the nucleus. Lipid-based NPs are biocompatible and stable particles; furthermore, there is no inflammatory response to these NPs when injected into the eye. However, there are also some disadvantages, such as lower gene expression when compared to the same transgenes delivered by viral vectors [174]. A new type of delivery system was recently developed by Trigueros and colleagues using gold nanoparticles (DNA-gold NPs), as these are relatively easy to generate and can be adapted to different shapes and sizes [206]. Gold is well-tolerated inside an organism and presents low rates of toxicity. However, they present a low clearance rate, thereby hampering their uptake in specific cells or tissues [207]. Therefore, Trigueros et al. further optimized this system for IRDs. In this study, they compared this new system with DNA-liposome complexes and demonstrated higher expression of a reporter gene. Their results showed that RPE cells responded differently to pristine-gold NPs and DNA-gold NPs, probably because of changes in the membrane-NP interface. Moreover, both types of NPs used different alternative endocytic ways for internalization, as indicated by their detection in different endosomal compartments. DNA-gold NPs were located in early endosomes that later will not mature into late endosome vesicles, resulting in an earlier expression of the reporter gene compared to pristine-gold NPs. This study was the first step to use non-cytotoxic gold NPs for an in vivo gene therapy to treat IRDs. However, some biological questions are still unclear, such as the internalization routes that are used and the exact nature of intracellular endosome trafficking. Furthermore, before using these particles as a delivery method in the retina, it is necessary to understand the complexity of cell–NP interaction, enhance the cargo delivery by avoiding degradative pathways (such as the lysosomal pathway) and ensure a constant expression of the therapeutic gene [206].

Finally, cationic lipids (liposomes) offer an alternative way to deliver DNA into the eye. In addition to the two most common ways of retinal delivery (see above), other delivery methods such as topical or intravenous administration have already reported positive results in eye diseases [174]. Liposomes however also present some limitations such as retinal toxicity and aggregation following administration [208]. Consequently, researchers developed a new type of liposome-PEGylated with perfluoropropane gas-that appears to be safer and more efficient for transfection but, as happened with other lipid-based vectors, presented a drop in gene expression four days after administration [174].

## 4. Other IRD Treatments

While gene-based therapies may stop or at least delay, the progression of the disease, other promising approaches that are less dependent of the genetic cause of the disease are also gaining momentum. This is for instance the case for stem cell-derived retinal cell transplantation (cell therapy) or the use of prosthetic implants.

### 4.1. Cell Therapy

Retinal cells, like other cells within the central nervous system, present a low regeneration potential. Therefore, cell therapies could be applied in those IRDs that present an advanced degeneration stage. The use of this type of therapy aims to result in an integration of exogenously delivered cells and subsequent re-activation of visual function [45]. Patient-derived somatic cells could be used to reprogram induced pluripotent stem cells (iPSCs) that subsequently could be differentiated to retinal precursor cells and introduced into the eye to replace either photoreceptors or supporting cells (e.g., RPE) that provide trophic and metabolic maintenance to prevent further degeneration of the remaining photoreceptors [209]. Genome editing tools (such as those described in Section 2.1.2) can be used to repair patient-specific mutations, to eventually transplant the corrected cells back to the patient [210]. The use of embryonic stem cells (ESCs) would not require this genetic modification and it has been demonstrated that these cells also have a high capacity to differentiate into retinal precursors [45,211]. However, the use of ESC is associated with ethical considerations not present with the use of iPSCs generated from the patients. Moreover, the transplantation of iPSC-derived retinal cells would avoid the risk of immune rejection after surgery [45].

Following up on the promising advances that demonstrated that stem cell-derived photoreceptor transplantation can restore rod- and cone-mediated vision [211,212,213,214], recent studies showed that these transplanted cells are not able to integrate well into non-degenerative host retinas. Instead, it seems that post-mitotic donor and host photoreceptors can exchange RNA and proteins, including rhodopsin [215,216]. The visual improvements measured after stem cell-derived photoreceptor transplantation could thus also be the result of endogenous photoreceptors that have taken up donor cell-derived proteins. Recently, it was demonstrated that cell integration as well as cytoplasmic transfer can occur but the relative contributions of each depend on the environment within the host retina [45,216].

Some cell therapies that are already tested in the clinic use hESC or hiPSC-derived RPE, for treating diseases such as AMD or Stardgardt disease [217,218,219,220]. For the study employing iPSC-derived RPE transplantation, a one-year follow-up analysis indicated that the transplantation did not generate any adverse effect and no immune response was induced, even in the absence of immunosuppression. One of the studies using hESC-derived RPE also reported an improvement of vision in patients with age-related macular degeneration as well as those with Stargardt disease [218]. Nevertheless, more studies are needed to provide reproducible protocols to generate iPSC-derived photoreceptor precursor cells. In addition, if such cells are transplanted after gene mutation repair, stringent quality controls of the iPSCs before and after genome editing are extremely important [45].

### 4.2. Retinal Prosthetic Implants

Inner retinal neurons largely retain their capability of signal transmission and are still present in advanced stages of retinal degradation. This fact encouraged the use of a stimulation mechanism (prosthetic implants) that is able to restore vision to some extent. Such a device would bypass the degraded photoreceptor layer and directly interact with the still functioning inner retinal neurons [84]. Retinal prostheses work as an integral system that contain an image acquisition device (which is integrated by thousands of light-sensitive microphotodies), an image processor, a stimulator chip and an electrode array [84,221]. In this system, light emanating from visible objects is converted by the microphotodies into little currents of hundreds of microelectrodes, which are directed onto remaining neurons within the neuronal network, the middle and the inner retina [221]. These systems have demonstrated a partial visual restoration, presenting the first evidence of this strategy in the field of vision [222]. To date, several stimulation modalities have been built [84] and four of them have obtained market approval for use in Europe and/or United States (Argus II, IRIS, IMS and AMS) [222]. These devices are classified according to their anatomical placement.

Epiretinal prostheses (Argus II, IRIS and EPI-IRET3) are implanted on the surface of the neurosensory retina, adjacent to nerve fibre and ganglion cell layers. Its location ensures certain advantages such as easy surgical delivery and safe heat dispersion [84,222]. Functionally, the stimulation is directly applied to the retinal ganglion cells, bypassing the residual intraretinal processing system, thereby inhibiting the capacity to mimic the physiological topographic organization. Besides this, as the epiretinal prostheses are close to passing axonal nerve fibres, ectopic visual perceptions from axonal stimulation can occur, thereby decreasing spatial resolution and confusing the intended stimulation pattern. As a note, the epiretinal prostheses have an external camera positioned outside the eye that provides power induction and a data signal that is transmitted to the intraocular simulator [84,221,222]. From these prostheses, Argus II is the most widely-used and it has been implanted in more than 250 patients to date, reporting encouraging results [223].

Subretinal prostheses (ASR, IMS/AMS, PRIMA and BSI) are placed between the degenerated photoreceptor layer and the RPE, such that the intrinsic signal processing capability of the retinal interneurons can be used optimally, generating vision similar to the one that is physiologically generated in the eye [84,222]. Moreover, the device is placed closer to the retina and is therefore favoured over the natural retinal signal amplification, requiring lower stimulation intensities. Unless the system has intrinsic photosensitivity and amplification capacity, it, like the epiretinal devices, requires a power source and a connection serving the delivery of data. Surgically, some studies have indicated that positioning these devices could be technically complicated because of the RPE adhesion caused by the retinal degeneration. Moreover, this surgical approach is less known and practiced outside routine retinal surgery [222]. To date, there are two methods to subretinal stimulation—one that employs a standard electrode array and another that uses a microphotodiode array (MPDA) that is present in all devices with exception of BSI (Boston retinal implant). This last one itself is able to capture light, allowing to avoid the use of cameras, while the visual scene is perceived by the lens on the array [84].

Suprachoroidal prostheses (STS and BVA) are located in the suprachoroidal space. This space is highly vascular and therefore there is a high risk of haemorrhage and fibrosis post-implantation. In comparison with the other two counterparts mentioned above, suprachoroidal devices are relatively far away from the retina. This implies that the design requires greater stimulation power to elicit visual perception [84,222]. Larger numbers are needed to establish solid conclusions about the efficacy of both suprachoroidal and transscleral implants in their present formats; however, results to date suggest greater limitations to these approaches compared to epiretinal or subretinal implants [84].

## 5. Concluding Remarks

The approval of Luxturna^TM^ as the first approved gene augmentation therapy for an ocular disease has provided an enormous impulse to the development of retinal therapeutics, both in academic centres as well as in industry. As summarized in this review, current developments range from gene augmentation, splice modulation, genome editing, optogenetics and compound therapies to cell replacement strategies and retinal prostheses. Patients with progressive vision loss are in need of treatment, to improve their quality of life by (partially) restoring vision or at least slow down or halt the progression of their diseases. Which strategy has the highest chance of being safe and efficacious depends on many factors, including the person’s genetic defect(s) and the stage of disease accompanied by the appearance of the retina. However, therapeutic development also requires appropriate cellular and/or animal models to test the efficacy of a given approach, as well as clinical endpoints to determine whether an improvement of therapeutic intervention can be measured. Only when fundamental and translational scientists, clinicians, funding agencies, patient organizations, industry and regulatory bodies join forces, we can fight these devastating conditions and provide hope and vision, for thousands of visually impaired individuals worldwide. 

## Figures and Tables

**Table 1:** Summary of clinical trials for inherited retinal diseases (IRDs) using DNA therapies.

Gene/Condition	Therapeutic Molecule	Clinical Trial Identifier	Status
***Gene Augmentation***			
*ABCA4*	SAR422459	NCT01367444	Recruiting
	SAR422459	NCT01736592	Enrolling by invitation
*CHM*	AAV2-hCHM	NCT02341807	Active, not recruiting
	AAV2-REP1	NCT03496012	Recruiting
	AAV2-REP1	NCT03507686	Recruiting
	AAV2-REP1	NCT02407678	Active, not recruiting
	rAAV2.REP1	NCT02671539	Active, not recruiting
	rAAV2.REP1	NCT02077361	**Completed**
	rAAV2.REP1	NCT01461213	**Completed**
	AAV2-REP1	NCT03584165	Enrolling by invitation
	AAV2-REP1	NCT02553135	**Completed**
*CNGA3*	AGTC-402	NCT02935517	Recruiting
	AAV2/8-hG1.7p.coCNGA3	NCT03758404	Recruiting
	rAAV.hCNGA3	NCT02610582	Active, not recruiting
*CNGB3*	rAAV2tYF-PR1.7-hCNGB3	NCT02599922	Recruiting
	AAV2/8-hCARp.hCNGB3	NCT03001310	Recruiting
	AAV-CNGB3	NCT03278873	Recruiting
*MERTK*	rAAV2-VMD2-hMERTK	NCT01482195	Recruiting
*MYO7A*	UshStat	NCT02065011	Enrolling by invitation
*PDE6B*	AAV2/5-hPDE6B	NCT03328130	Recruiting
*RPE65*	AAV OPTIREP	NCT02946879	Recruiting
	rAAV2-CBSB-hRPE65	NCT00481546	Active, not recruiting
	AAV2-hRPE65v2-	NCT00516477	Active, not recruiting
	AAV2-hRPE65v2	NCT00999609	Active, not recruiting
	AAV2-hRPE65v2	NCT01208389	Active, not recruiting
	AAV2-hRPE65v2	NCT03602820	Active, not recruiting
	tgAAG76 (rAAV 2/2.hRPE65p.hRPE65)	NCT00643747	**Completed**
	rAAV2-CB-hRPE65	NCT00749957	**Completed**
	rAAV2-hRPE65	NCT00821340	**Completed**
	rAAV2/4.hRPE65	NCT01496040	**Completed**
	AAV RPE65	NCT02781480	**Completed**
*RLBP1*	CPK850	NCT03374657	Recruiting
*RPGR*	AAV8-RPGR	NCT03116113	Recruiting
	AAV2/5-hRKp.RPGR	NCT03252847	Recruiting
	rAAV2tYF-GRK1-RPGR	NCT03316560	Recruiting
*RS1*	AAV8-scRS/IRBPhRS	NCT02317887	Recruiting
	rAAV2tYF-CB-hRS1	NCT02416622	Active, not recruiting
***Genome editing***			
*CEP290*	EDIT-101 (AGN-151587)	NCT03872479	Recruiting
***Optogenetics***			
Advanced RP	RST-001	NCT02556736	Recruiting
Non-syndromic RP Retinitis Pigmentosa	GS030-DP	NCT03326336	Recruiting

Black and gray are just used to indicate the different sectiond and can be considered as headers. Gene or disease condition (first column), therapeutic molecule (second column), clinical trial identifier (third column) and the current status of the trial (last column) are indicated. Completed trials are highlighted in bold. Data obtained from https://clinicaltrials.gov/. RP: retinitis pigmentosa.

**Table 2:** List of clinical trials for IRDs using RNA therapies.

Gene	Therapeutic Molecule	Clinical Trial Identifier	Status
*CEP290*	QR-110	NCT03140969	Active, not recruiting
	QR-110	NCT03913130	Recruiting
	QR-110	NCT03913143	Recruiting
*USH2A*	QR-421a	NCT03780257	Recruiting

Black is just used to indicate the different sectiond and can be considered as headers. Gene (first column), therapeutic molecule (second column), clinical trial identifier (third column) and current status of the trial (last column) are indicated. Data obtained from https://clinicaltrials.gov/.

**Table 3:** Summary of clinical trials for IRDs using compound therapies.

Gene	Therapeutic Molecule	Clinical Trial Identifier	Status
*ABCA4*	ALK-001	NCT02402660	Recruiting
	Zimura	NCT03364153	Active, not recruiting
	Emuxustat	NCT03772665	Recruiting
	Emuxustat	NCT03033108	**Completed**
*RPE65*	QLT091001	NCT01014052	**Completed**
	QLT091001	NCT01521793	**Completed**
	QLT091001	NCT01543906 *	**Completed**
*RS1*	Dorzolamide 2% TID or brinzolamide 1% TID	NCT02331173	**Completed**

Black is just used to indicate the different sectiond and can be considered as headers. Gene (first column), therapeutic molecule (second column), clinical trial identifier (third column) and current status of the trial (last column) are indicated. Completed trials are highlighted in bold. Data obtained from https://clinicaltrials.gov/. * Of note, Trial NCT01543906 describes the use of QLT091001 in patients with a dominant *RPE65* mutation (unlike most *RPE65* mutations inherited in an autosomal recessive manner).
